# Locally advanced breast cancer: breast-conserving surgery and other factors linked to overall survival after neoadjuvant treatment

**DOI:** 10.3389/fonc.2023.1293288

**Published:** 2023-11-06

**Authors:** Gabriela Bezerra Nobrega, Bruna Salani Mota, Gabriela Boufelli de Freitas, Jonathan Yugo Maesaka, Rosa Maria Salani Mota, Rodrigo Goncalves, Angela Francisca Trinconi, Marcos Desidério Ricci, José Roberto Piato, José Maria Soares-Jr, Edmund Chada Baracat, José Roberto Filassi

**Affiliations:** ^1^ Disciplina de Ginecologia, Departamento de Obstetrícia e Ginecologia, Hospital das Clínicas, Faculdade de Medicina, Universidade de São Paulo, São Paulo, Brazil; ^2^ Setor de Mastologia, Divisão de Ginecologia, Instituto do Câncer do Estado de São Paulo, Hospital das Clínicas, Faculdade de Medicina, Universidade de São Paulo, São Paulo, Brazil; ^3^ Departamento de Estatística e Matemática Aplicada, Universidade Federal do Ceará, Fortaleza, Brazil

**Keywords:** breast neoplasms, neoadjuvant therapy, local disease, segmental mastectomy, breast-conserving surgery, survival rate, locally advanced breast cancer

## Abstract

**Background:**

Recent data suggest that breast-conserving surgery (BCS) may positively impact overall survival (OS) in early breast cancer. However, the role of BCS in locally advanced breast cancer (LABC) following neoadjuvant therapy (NAT) remains uncertain.

**Methods:**

We conducted a retrospective cohort study involving 530 LABC patients who underwent surgery after NAT between 2010 and 2015. Outcomes examined included OS, distant recurrence rates (DRR), and loco-regional recurrence rates (LRRs).

**Results:**

Among the 927 breast cancer patients who received NAT, 530 were eligible for our study. Of these, 24.6% underwent BCS, while 75.4% underwent mastectomy (MS). The median follow-up duration was 79 months. BCS patients exhibited a higher pathological complete response (PCR) rate compared to those who underwent MS (22.3% vs. 10%, *p* < 0.001). The 6-year OS rates for BCS and MS were 81.5% and 62%, respectively (*p* < 0.000). In multivariate OS analysis, MS was associated with worse outcomes (OR 1.678; 95% CI 1.069–2.635; *p* = 0.024), as was body mass index (BMI) (OR 1.031; 95% CI 1.006–1.058; *p* = 0.017), and stage IIIB or IIIC (OR 2.450; 95% CI 1.561–3.846; *p* < 0.000). Conversely, PCR (OR 0.42; 95% CI 0.220–0.801; *p* = 0.008) was associated with improved survival. DRR was significantly lower in BCS (15.4%) compared to MS (36.8%) (OR 0.298; 95% CI 0.177–0.504). LRRs were comparable between BCS (9.2%) and MS (9.5%) (OR 0.693; 95% CI 0.347–1.383).

**Conclusion:**

Our findings suggest that BCS is oncologically safe, even for patients with large lesions, and is associated with superior OS rates compared to MS. Additionally, lower BMI, lower pretreatment stage, and achieving PCR were associated with improved survival outcomes.

## 1 Introduction

Locally advanced breast cancer (LABC), categorized as stage IIB or III (1), poses a significant health challenge, accounting for 14.23 deaths per 100,000 Brazilian women in 2019 (2). Approximately 25% of these cases are diagnosed at stage III breast cancer. Contemporary treatment strategies for LABC involve a multimodal approach that combines systemic and local treatments (3). However, one critical aspect of this treatment regimen that remains uncertain is the choice of surgical intervention following neoadjuvant therapy (NAT), especially in cases that initially had a mastectomy (MS) indication. Despite the apparent necessity of complete initial tumor bed removal, there is a growing inclination toward adopting more conservative surgical approaches, introducing a notable gap in the literature regarding the oncological safety of such a shift.

Over the past decade, the scientific community has witnessed a discourse surrounding the comparison of breast-conserving surgery (BCS) versus MS. A meta-analysis, conducted in 2022 and encompassing over 1,500,000 patients, albeit excluding those who underwent NAT, suggested that BCS yielded superior overall survival (OS) outcomes compared to MS (4). Conversely, another meta-analysis, focusing on studies with both neoadjuvant and adjuvant treatments, as conducted by the Early Breast Cancer Trialists’ Collaborative Group (EBCTCG) (5), revealed that while BCS was associated with higher loco-regional recurrence rates (LRRs), it did not significantly impact OS. In a separate study, Simons et al. reported that BCS contributed to increased OS compared to MS in an unadjusted model (6). Gwark et al. corroborated this observation demonstrating the same outcome in both unadjusted and adjusted analyses (7). Nonetheless, most of these trials included early-stage tumors, with a dearth of data pertaining to LABC.

Considering the uncertainties and the contrasting findings in the existing literature, we have undertaken this study to ascertain whether BCS has a discernible impact on OS and LRR in patients with LABC who have undergone NAT. This investigation aims to contribute valuable insights into the optimal surgical management of LABC, particularly in cases where BCS may present a viable alternative to more radical procedures like MS.

## 2 Materials and methods

### 2.1 Patients

This study conducted a retrospective cohort analysis in accordance with the Strengthening the Reporting of Observational Studies in Epidemiology (STROBE) guidelines (8). It encompassed all consecutive patients diagnosed with LABC who underwent NAT at Instituto do Câncer de São Paulo (ICESP) between January 2010 and December 2015. Inclusion criteria encompassed women with LABC considered suitable candidates for MS for breast cancer treatment before NAT. The sequence of treatment commenced with NAT, involving chemotherapy or endocrine therapy, followed by surgical intervention. Patients were excluded if they exhibited contraindications to radiotherapy (RT), presented with distant metastasis at the time of diagnosis, or had a history of multiple malignancies.

Patient data extracted from medical records included age at diagnosis, body mass index (BMI), type of surgery, type of NAT, tumor size, pathological stage, clinical stage, RT, histological subtype, histological grade, nuclear grade, and molecular subtype tumor, determined based on the expression of estrogen receptor (ER), progesterone receptor (PR), and human epidermal growth factor receptor-2 (HER2). Immunohistochemical methods were employed to evaluate ER, PR, and HER2 status. ER and PR positivity was established when more than 1% of cells displayed positive staining. HER2 overexpression analysis categorized cases graded 0 or 1+ as negative and 3+ as positive. For cases graded 2+, fluorescence *in situ* hybridization was conducted. Tumor staging followed the TNM classification, 7th edition (9), with LABC defined as encompassing stages IIB and III. NAT and RT adhered to the National Comprehensive Cancer Network (NCCN) guidelines (10).

Pathological complete response (PCR) was defined as ypT0 ypN0. Patients underwent regular follow-up every 3–6 months for the initial 5 years and annually thereafter. Disease relapse and metastasis were detected based on clinical examinations conducted during follow-up visits, along with yearly mammography. Additional assessments, including chest computed tomography, bone scans, and liver ultrasonography, were performed in response to abnormal clinical findings. Loss of follow-up was defined as an interval exceeding 2 years until the last medical appointment. This study obtained approval from the institutional ethics committee (NP 856/2015).

### 2.2 Outcomes

The primary outcome was OS, defined as the time from surgery to death attributed to any cause. Secondary outcomes included the assessment of distant recurrence rates (DRRs) and LRRs in relation to the two surgical groups, namely, BCS and MS. LRR was specifically defined as the initial recurrence manifestation in the breast, ipsilateral axilla, and ipsilateral supraclavicular region, while DRR pertained to the first occurrence of distant metastasis.

### 2.3 Statistical analysis

Measures of central tendency, such as mean and median, along with measures of dispersion, were employed to evaluate continuous variables. The data’s distribution characteristics were assessed using the Kolmogorov–Smirnov and Shapiro–Wilk tests. To compare the distribution of quantitative variables across two or more groups, we employed the chi-square and Mann–Whitney tests. Continuous variables were assessed using the unpaired Student’s ***t***-test. The odds ratios were estimated utilizing Poisson regression.

Time-to-event outcomes were analyzed through the Kaplan–Meier survival function, and differences between groups were assessed with the Log-Rank test. To assess the independent prognostic effect of the surgical method on OS and disease-free survival (DFS), while accounting for various prognostic factors, we employed the Cox proportional-hazards model.

Statistical analyses were conducted using the SPSS software version 20.0. A significance level of *p* = 0.05 was utilized for all statistical tests, indicating a 5% threshold for statistical significance.

## 3 Results

### 3.1 Patient characteristics

Initially, a total of 927 women with breast cancer who underwent NAT were evaluated. A total of 530 eligible patients with LABC who underwent NAT and subsequent surgery, adhering to the eligibility criteria, were included in this study. Among these patients, 506 (95.4%) received neoadjuvant chemotherapy, and 24 (4.5%) received neoadjuvant endocrine therapy. The average age of the patient population was 52.7 ± 1.2 years, with an age range spanning from 23 to 95 years.

Out of the 530 patients, 138 (26.1%) had stage IIB, while 391 (73.9%) had stage III breast cancer. The histological subtypes were distributed as follows: 201 (37.9%) luminal HER2 negative, 189 (35.6%) triple-negative, 71 (13.5%) luminal HER2 positive, and 69 (13.1%) HER2 positive. PCR was observed in 13.0% (69 patients) of cases. Regarding the choice of surgery, 130 patients (24.6%) underwent BCS, while 400 patients (75.4%) underwent MS.

Comparing the BCS and MS groups, statistically significant differences were noted. The BCS group consisted of older patients (*p* < 0.001), individuals with earlier-stage disease (*p* < 0.001), and higher BMI (*p* < 0.001). Additionally, the BCS group had a higher proportion of post-menopausal patients, multiparous individuals, and those who underwent sentinel lymph node (SLN) biopsy (*p* < 0.001). Notably, the PCR rate was significantly higher in the BCS group, with 22.3% (29 patients), compared to 10% (40 patients) in the MS group (*p* < 0.001). Details are provided in **Table 1**.

**Table 1:** Clinical and pathological characteristics in univariate analysis of patients undergoing conservative surgery when compared to mastectomy.

	Breast-conserving surgery *N* = 130 (24.60%)		Mastectomy *N* = 400 (75.40%)		OR	95% CI	*p*
**Age (years)** **(median ± SD)**	55.0 ( ± 11.7)		50.6 ( ± 11.9)				0.000
**BMI (kg/m²)** **(median ± SD)**	30.3 (± 5.9)		28.3 ( ± 5.5)				0.001
Menopause							
No	52	40.0%	202	50.5%	1	–	0.043
Yes	78	60.0%	198	49.5%	1.530	1.024–2.287	
Nulliparity							
No	113	87.6%	335	84.2%	1	–	0.396
Yes	16	12.4%	63	15.8%	0.753	0.418–1.356	
Stage							
IIB	55	42.3%	83	20.8%	1	–	0.000
IIIA	56	43.1%	166	41.50%	0.509	0.323–0.803	
IIIB or IIIC	19	14.6%	151	37.80%	0.190	0.106–0.341	
Biological subtype							
ER (–) PR (–) HER2 (–)	53	40.8%	136	34.0%	1	–	0.320
ER (-) PR (-) HER2 (+)	12	9.2%	57	14.3%	0.540	0.269–1.086	
ER (+) PR (+) HER2 (+)	15	11.5%	56	14.0%	0.687	0.358–1.320	
ER (+) PR (+) HER2 (-)	50	38.5%	151	37.8%	0.850	0.541–1.333	
Neoadjuvant treatment							
Chemotherapy	122	93.8%	384	96.0%	1	–	0.332
Endocrine therapy	8	6.2%	16	4.0%	1.574	0.657–3.767	
Pathological complete response							
No	101	77.7%	360	90.0%	1	–	0.001
Yes	29	22.3%	40	10.0%	2.584	1.526–4.375	
Axillary surgery							
Sentinel lymph node	18	13.8%	19	4.8%	1	–	0.001
Axillary dissection	112	86.2%	381	95.3%	0.310	0157–0.611	
Radiotherapy							
No	4	3.1%	19	4.8%	1	–	0.62
Yes	126	96.9%	381	95.3%	1.571	0.525–4.704	
Recurrence							
No	98	75.4%	215	53.8%	1	–	0.000
Yes	32	24.6%	185	46.3%	0.379	0.243–0.592	
Systemic recurrence	20	15.4%	147	36.8%	0.298	0.177–0.504	
Local recurrence	12	9.2%	38	9.5%	0.693	0.347–1.383	
Ki-67 (%)							
≤30	54	41.5%	179	45.4%	1	–	0.477
>30	76	58.5%	215	54.6%	1.172	0.784–1.750	
Death							
No	106	81.5%	248	62.0%	1	–	0.000
Yes	24	18.5%	152	38.0%	0.394	0.242–0.641	

BMI, body mass index; ER, estrogen receptor; PR, progesterone receptor; HER2, tissue human epidermal growth factor receptor-2.

Type of NAT, RT, histological subtype, molecular profile, and Ki-67 values were similar between the two surgical groups. A total of 65 patients (12.2%) were lost to follow-up.

### 3.2 Breast surgical management

Univariate analysis revealed clinical factors favoring BCS, including older age, higher BMI, menopause, lower staging, PCR, and a more conservative axillary approach, such as SLN biopsy (**Table 1**).

In the multivariate analysis assessing the factors influencing the choice of BCS over MS, it was observed that lower stage (IIB: OR 1.00; 3A: OR 0.49; 95% CI 0.302–0.794; *p* = 0.004; IIIB/IIIC: OR 0.15; 95% CI 0.083–0.286; *p* < 0.001), PCR (OR 2.49; 95% CI 1.403–4.426; *p* = 0.002), age (55.0 ± 11.7 versus 50.6 ± 11.9; OR 1.034; 95% CI 1.015–1.053; *p* < 0.001), and BMI (30.3 ± 5.9 versus 28.3 ± 5.5; OR 1.069; 95% CI 1.029–1.110; *p* = 0.001) were independent factors associated with the choice of BCS (**Table 2**).

**Table 2:** Multivariate analysis of patients undergoing breast-conserving surgery compared to mastectomy.

	OR	95% CI	*p*
Stage			
IIB	1	–	
IIIA	0.490	0.302–0.794	0.004
IIIB or IIIC	0.154	0.083–0.286	0.000
Pathological complete response			
Yes	2.492	1.403–4.426	0.002
No	1	–	
Age	1.034	1.015–1.053	0.000
Body mass index	1.069	1.029–1.110	0.001

### 3.3 Local and distant recurrence

The median follow-up period for both the BCS and MS groups was similar, at 80 and 78 months, respectively (*p* = 0.89). Over the course of the follow-up, 217 patients (41%) experienced systemic and/or loco-regional recurrence. A statistically significant difference in DRR was observed between the BCS and MS groups. Specifically, DRR was 15.4% (20/130) for BCS and 36.8% (147/400) for MS (OR: 0.298; 95% CI: 0.177–0.504). However, LRR did not exhibit a statistically significant difference between the two groups, with LRR rates of 9.2% (12/130) for BCS and 9.5% (38/400) for MS (OR: 0.693; 95% CI: 0.347–1.383) (**Table 3**).

**Table 3:** Recurrence rates.

	Breast-conserving surgery *N* = 130 (%)	Mastectomy *N* = 400 (%)	Total patients *N* = 530 (%)	OR	95% CI	*p*
**Any recurrence**	32 (24.6)	185 (46.3)	217 (40.9)	0.379	0.243–0.592	0.001
**Systemic recurrence**	20 (15.4)	147 (36.8)	167 (31.5)	0.298	0.177–0.504	
**Local recurrence**	12 (9.2)	38 (9.5)	50 (9.4)	0.693	0.347–1.383	

### 3.4 Overall survival

**Figure 1:**
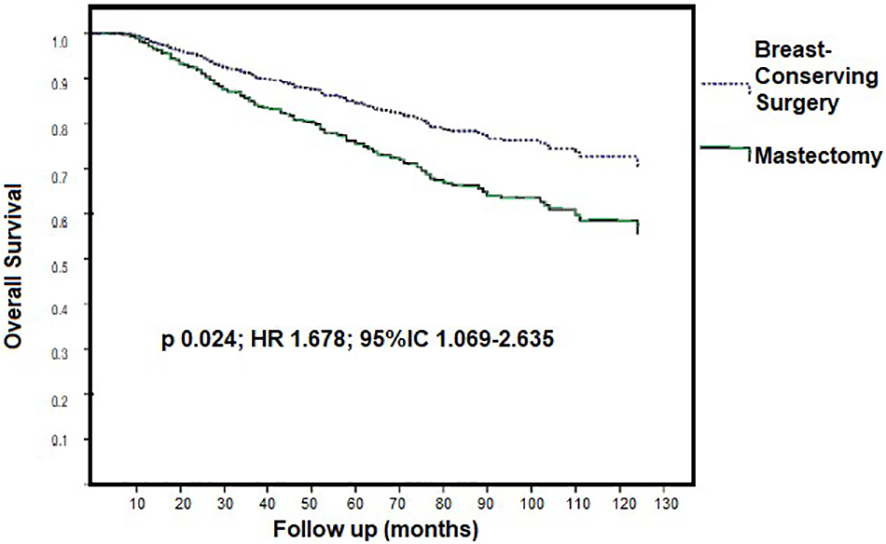
Kaplan-Meier curves for survival according to surgery after multidimesional analysis.

The 6-year OS rates for patients who underwent BCS and MS were 81.5% and 62%, respectively (log-rank, p < 0.001) (**Figure 1**). Univariate analysis revealed that MS was significantly associated with worse OS compared to BCS. Additionally, menopausal status, BMI, PCR, staging, and breast and axillary surgery were factors associated with lower OS. Following multivariate analysis, as presented in **Table 4**, MS remained a significant predictor of worse OS (OR 1.678; 95% CI 1.069–2.635; p = 0.024), along with BMI (OR 1.031; 95% CI 1.006–1.058; *p* = 0.017) and staging IIIB or IIIC (OR 2.450; 95% CI 1.561–3.846; *p* < 0.001). Conversely, PCR was associated with improved OS (OR 0.42; 95% CI 0.220–0.801; *p* = 0.008).

**Table 4:** Multivariate analysis of factors that significantly differs in overall survival.

	*p*	HR	95% CI
Body mass index	0.017	1.031	1.006–1.058
Surgery			
Breast-conserving surgery		1	–
Mastectomy	0.024	1.678	1.069–2.635
Stage			
IIB	0.000	1	–
IIIA		1.534	0.978–2.407
IIIB or IIIC		2.450	1.561–3.846
Pathological complete response			
Yes	0.008	0.420	0.220–0.801
No		1	–

## 4 Discussion

Our study demonstrates that BCS following NAT is oncologically safe and an independent factor for improving long-term OS in patients initially considered candidates for MS due to locally advanced tumors.

In early breast cancer, the Veronesi trial (11) established the efficacy of BCS combined with RT as a viable option with comparable OS to MS. A recent meta-analysis, comprising 30 studies and over 1.5 million patients who underwent upfront surgery, reported that BCS plus RT yielded superior OS rates compared to MS, with a 36% improvement (confidence interval ranging from 26% to 45%). Notably, this difference became more pronounced when focusing solely on cohort studies, reaching a 46% improvement in OS. However, after 10 years, these differences tended to disappear. When considering only six clinical trials with 3,933 participants, there was no significant difference in terms of local recurrence in the meta-analysis (4).

Following the introduction of NAT, BCS also emerged as a feasible option for LABC. When comparing BCS to MS in patients who received neoadjuvant chemotherapy, studies consistently demonstrated no significant differences in OS or LRR (12–14). A meta-analysis conducted by Sun et al. (14), incorporating five studies (12, 15–18) with a total of 1,114 patients, indicated that BCS was a safe surgical approach after NAT for LABC and was associated with improved OS (OR 2.12; 95% CI 1.51–2.98, *p* < 0.01) compared to the MS group (14). However, it is important to note that, upon closer examination, only approximately 8.5% (*N* = 95/1,114) of patients had T3 tumors, and approximately 26.8% (***N*** = 299/1,114) were classified as stage III (12, 15–17). One study included 119 patients (11.5%) and reported a median tumor size of approximately 41 mm (18). Although these data suggest similar survival rates, the inclusion criteria in these studies were heterogeneous (14).

Gwark et al. published a retrospective cohort study involving 1,641 patients who received NAT before surgery and reported significantly better OS in the BCS plus RT group. Initially, most patients had T2 stage tumors (61.9%, *N* = 1,017). However, after propensity score matching, the study focused on 378 patients, including 198 with T2 tumors, 138 with T3 tumors, and 23 with T4 tumors (7). Our dataset differs in that it includes a higher proportion of patients diagnosed with LABC, comprising 74% (*N* = 392) of stage III and 26% (*N* = 138) of patients diagnosed with stage IIB breast cancer.

The criteria for selecting BCS following NAT for LABC mirror those applied in upfront surgery, including the importance of maintaining a favorable tumor–breast relationship (19). However, a degree of uncertainty exists regarding the extent of primary tumor area removal after NAT. According to recent literature, the post-treatment tumor size serves as the reference point when determining the appropriate surgical approach (14).

In our cohort study, several factors were associated with an increased likelihood of choosing BCS after NAT. These factors included an earlier clinical stage, a higher rate of pathological clinical responses (PCRs), older age, and a higher BMI. Specifically, BCS was 50% more likely to be chosen in stage IIB cases. When PCR was achieved, the chances of opting for BCS increased 2.5 times. Additionally, women older than 55 and those with a BMI greater than 30 had 3% and 6% higher chances of choosing BCS, respectively. This can be attributed to the fact that individuals with smaller tumors both before and after NAT, and a higher BMI, often have a more favorable tumor–breast relationship.

Regarding OS after NAT, BCS, lower BMI, lower pre-NAT staging, and PCR were all associated with better OS rates. A 2017 meta-analysis involving 3,531 participants (1,465 in the BCS arm and 2,066 in the MS arm) found that BCS was a safe option for LABC patients who showed an excellent response to NAT. This analysis reported a nearly 50% lower risk of distant recurrence, with a real effect protection ranging from 42% to 63%, and a twofold higher rate of OS and DFS in the BCS group (14). These findings align with our own, indicating that the BCS group exhibited improved OS and fewer systemic recurrences.

In contrast, the EBCTCG meta-analysis described a higher LRR rate for BCS without an increase in breast cancer-specific mortality. It is essential to note the heterogeneity between studies in the EBCTCG analysis, as it included neoadjuvant and adjuvant treatments, and some patients received only RT as local treatment (5). Our results are more in line with recently published studies (6, 7). Gwark et al. demonstrated a 14% absolute improvement in OS in the BCS plus RT group compared to MS in patients who underwent NAT and surgery (7). Since RT can significantly impact OS, it is noteworthy that in our dataset, there were no significant differences in RT rates between the BCS and MS groups. This is because all patients in our dataset had prior indications for RT, given their LABC diagnosis.

It is well-established that a higher BMI increases the risk of breast cancer in women, and it is estimated that approximately 1.4 billion people will be obese by 2035 (20). Patel et al. (21) established a significant causal link between BMI and OS in obese breast cancer patients, particularly those with hormone receptor-positive tumors, which were associated with shorter survival rates. A systematic study in 2014 showed that obesity increased breast cancer mortality, with relative risks (RRs) of 1.41 (95% CI: 1.29–1.53) for obese individuals (BMI >30.0) and 1.07 (95% CI: 1.02–1.12) for overweight individuals (BMI 25.0–30.0). The risk of death rose proportionally with BMI. For every 5 kg/m**^2^** increase in BMI before diagnosis, the risk of overall death and breast cancer-specific mortality increased by 17% and 18% before and after menopause, respectively (22). These findings further support our study’s conclusion that higher BMI is associated with an increased risk of mortality.

We observed that initial staging IIIB or IIIC breast cancer was a significant risk factor for death, increasing the risk by more than 2.4 times compared to staging IIB. This finding aligns with a 1988 article by Hortobagyi et al. (23), which evaluated only stage III patients, including those who had and had not received neoadjuvant chemotherapy. Large tumor sizes may not achieve a PCR, which has been shown to be associated with higher mortality. Interestingly, Meyers et al. (24) did not associate pretreatment stage with a worse prognosis for LRR. They suggest that the post-treatment stage is more related to LRR than the pretreatment stage. It is important to note that they did not analyze OS.

In our dataset, the rate of PCR had the most significant influence on OS. We defined PCR as ypT0 ypN0, which may have impacted our results. According to Cortazar et al.’s 2014 meta-analysis (25), the frequency of PCR varied depending on the definition: 22% of patients achieved ypT0/is, 18% achieved ypT0/is ypN0, and 13% achieved ypT0 ypN0. However, all definitions consistently resulted in an increase in both OS and DFS. Patients who had a favorable response to NAT, such as achieving PCR or a decreased tumor size after NAT, were better candidates for BCS, as confirmed by another meta-analysis conducted in 2017 (14).

While this result should be interpreted cautiously for patients with LABC, as it is from a tertiary single-center investigation with a 5-year follow-up, it underscores the treatment uniformity and provides compelling evidence that BCS after NAT is a favorable option for women with LABC, leading to improved OS. Furthermore, considering the global obesity pandemic, our research highlights the significance of effective weight management in influencing oncological prognosis. It is worth noting that only approximately 12% of the patients were lost to follow-up.

Nonetheless, there are limitations associated with our study. As a retrospective cohort design, potential biases such as selection bias, an imbalance of prognostic factors, and reporting bias may exist, potentially affecting internal validity. To address these limitations, we performed a multivariable analysis. Despite these drawbacks, our cohort study has robust external validity, and its findings may be generalized to this breast cancer population. Moreover, our study can serve as a basis for other breast cancer study groups to plan clinical trials to further investigate this question.

In conclusion, our study suggests that BCS following NAT is oncologically safe and improves long-term survival for women with LABC. Additionally, it underscores the importance of maintaining a normal BMI, which significantly enhances a patient’s likelihood of survival. Based on these findings, patients receiving NAT should consider advocating for BCS when feasible and implementing weight-maintenance strategies that can enhance their quality of life and survival.

## Data availability statement

The original contributions presented in the study are included in the article/supplementary material. Further inquiries can be directed to the corresponding author.

## Ethics statement

The studies involving humans were approved by Comitê de Ética do departamento de Obstetrícia e Ginecologia da Faculdade de Medicina da Universidade de São Paulo, São Paulo, Brazil. The studies were conducted in accordance with the local legislation and institutional requirements. The ethics committee/institutional review board waived the requirement of written informed consent for participation from the participants or the participants’ legal guardians/next of kin because It is a retrospective study with analysis of data from medical records.

## Author contributions

GN: Conceptualization, Data curation, Investigation, Methodology, Project administration, Writing – original draft, Writing – review & editing. BM: Data curation, Investigation, Methodology, Project administration, Software, Supervision, Writing – original draft, Writing – review & editing. GF: Data curation, Investigation, Supervision, Writing – review & editing. JM: Data curation, Investigation, Supervision, Writing – review & editing. RM: Formal Analysis, Software, Writing – review & editing. RG: Methodology, Supervision, Writing – review & editing. AT: Supervision, Visualization, Writing – review & editing. MR: Supervision, Visualization, Writing – review & editing. JP: Methodology, Supervision, Validation, Writing – review & editing. JJ: Supervision, Validation, Writing – review & editing. EB: Supervision, Visualization, Writing – review & editing. JF: Investigation, Project administration, Supervision, Validation, Visualization, Writing – review & editing.

## References

[1] World Health Organization. Breast Cancer: early diagnosis and screening (2019). Available at: https://www.who.int/cancer/prevention/diagnosis-screening/breast-cancer/en/.

[2] INCA. Dados e números sobre o câncer de mama (2021). Available at: https://www.as.saude.ms.gov.br/wp-content/uploads/2021/11/Dados_e_numeros_site_cancer_mama_2021-1.pdf.

[3] Simon SD Bines J Werutsky G Nunes JS Pacheco FC Segalla JG. Characteristics and prognosis of stage I-III breast cancer subtypes in Brazil: The AMAZONA retrospective cohort study. Breast (2019) 44:113–9. doi: 10.1016/j.breast.2019.01.008 30738289

[4] De la Cruz Ku G Karamchandani M Chambergo-Michilot D Narvaez-Rojas AR Jonczyk M Príncipe-Meneses FS. Does Breast-Conserving Surgery with Radiotherapy have a Better Survival than Mastectomy? A Meta-Analysis of More than 1,500,000 Patients. Ann Surg Oncol (2022) 29(10):6163–88. doi: 10.1245/s10434-022-12133-8 35876923

[5] Early Breast Cancer Trialists' Collaborative Group (EBCTCG). Long-term outcomes for neoadjuvant versus adjuvant chemotherapy in early breast cancer: meta-analysis of individual patient data from ten randomised trials. Lancet Oncol (2018) 19(1):27–39. doi: 10.1016/S1470-2045(17)30777-5 29242041 PMC5757427

[6] Simons JM Jacobs JG Roijers JP Beek MA Boonman-de Winter LJM Rijken AM. Disease-free and overall survival after neoadjuvant chemotherapy in breast cancer: breast-conserving surgery compared to mastectomy in a large single-centre cohort study. Breast Cancer Res Treat (2021) 185(2):441–51. doi: 10.1007/s10549-020-05966-y PMC7867515 33073303

[7] Gwark S Kim HJ Kim J Chung IY Ko BS Lee JW. Survival after breast-conserving surgery compared with that after mastectomy in breast cancer patients receiving neoadjuvant chemotherapy. Ann Surg Oncol (2023) 30(5):2845–53. doi: 10.1245/s10434-022-12993-0 36577865

[8] Cuschieri S. The STROBE guidelines. Saudi J Anaesth. (2019) 13(Suppl 1):S31–S4. doi: 10.4103/sja.SJA_543_18 PMC6398292 30930717

[9] Edge SB Compton CC. The American Joint Committee on Cancer: the 7th edition of the AJCC cancer staging manual and the future of TNM. Ann Surg Oncol (2010) 17(6):1471–4. doi: 10.1245/s10434-010-0985-4 20180029

[10] (NCCN). NCCN. NCCN clinical practice guidelines in oncology (NCCN guidelines®). Breast cancer version 4.2023 (2023). Available at: https://www.nccn.org/professionals/physician_gls/pdf/breast.pdf.

[11] Veronesi U Cascinelli N Mariani L Greco M Saccozzi R Luini A. Twenty-year follow-up of a randomized study comparing breast-conserving surgery with radical mastectomy for early breast cancer. N Engl J Med (2002) 347(16):1227–32. doi: 10.1056/NEJMoa020989 12393819

[12] Cho JH Park JM Park HS Park S Kim SI Park BW. Oncologic safety of breast-conserving surgery compared to mastectomy in patients receiving neoadjuvant chemotherapy for locally advanced breast cancer. J Surg Oncol (2013) 108(8):531–6. doi: 10.1002/jso.23439 24115142

[13] Bleicher RJ Ruth K Sigurdson ER Daly JM Boraas M Anderson PR. Breast conservation versus mastectomy for patients with T3 primary tumors (>5 cm): A review of 5685 medicare patients. Cancer (2016) 122(1):42–9. doi: 10.1002/cncr.29726 PMC4707052 26479066

[14] Sun Y Liao M He L Zhu C. Comparison of breast-conserving surgery with mastectomy in locally advanced breast cancer after good response to neoadjuvant chemotherapy: A PRISMA-compliant systematic review and meta-analysis. Med (Baltimore). (2017) 96(43):e8367. doi: 10.1097/MD.0000000000008367 PMC5671859 29069026

[15] Schwartz GF Birchansky CA Komarnicky LT Mansfield CM Cantor RI Biermann WA. Induction chemotherapy followed by breast conservation for locally advanced carcinoma of the breast. Cancer (1994) 73(2):362–9. doi: 10.1002/1097-0142(19940115)73:2<362::AID-CNCR2820730221>3.0.CO;2-L 8293401

[16] Sweeting RS Klauber-Demore N Meyers MO Deal AM Burrows EM Drobish AA. Young women with locally advanced breast cancer who achieve breast conservation after neoadjuvant chemotherapy have a low local recurrence rate. Am Surg (2011) 77(7):850–5. doi: 10.1177/000313481107700718 PMC4167782 21944346

[17] Levy A Borget I Bahri M Arnedos M Rivin E Vielh P. Loco-regional control after neo-adjuvant chemotherapy and conservative treatment for locally advanced breast cancer patients. Breast J (2014) 20(4):381–7. doi: 10.1111/tbj.12277 24890310

[18] Barranger E Antomarchi J Chamorey E Cavrot C Flipo B Follana P. Effect of neoadjuvant chemotherapy on the surgical treatment of patients with locally advanced breast cancer requiring initial mastectomy. Clin Breast Cancer. (2015) 15(5):e231–5. doi: 10.1016/j.clbc.2015.03.001 25887149

[19] Tewari M Krishnamurthy A Shukla HS. Breast conservation in locally advanced breast cancer in developing countries: wise or waste. Surg Oncol (2009) 18(1):3–13. doi: 10.1016/j.suronc.2008.07.004 18722112

[20] The Lancet Diabetes Endocrinology. Let's talk about obesity. Lancet Diabetes Endocrinol (2023) 11(4):217. doi: 10.1016/S2213-8587(23)00059-1 36878240

[21] Patel V James M Frampton C Robinson B Davey V Timmings L. Body mass index and outcomes in breast cancer treated with breast conservation. Int J Radiat Oncol Biol Phys (2020) 106(2):369–76. doi: 10.1016/j.ijrobp.2019.09.049 31678226

[22] Chan DSM Vieira AR Aune D Bandera EV Greenwood DC McTiernan A. Body mass index and survival in women with breast cancer-systematic literature review and meta-analysis of 82 follow-up studies. Ann Oncol (2014) 25(10):1901–14. doi: 10.1093/annonc/mdu042 PMC4176449 24769692

[23] Hortobagyi GN Ames FC Buzdar AU Kau SW McNeese MD Paulus D. Management of stage III primary breast cancer with primary chemotherapy, surgery, and radiation therapy. Cancer (1988) 62(12):2507–16. doi: 10.1002/1097-0142(19881215)62:12<2507::AID-CNCR2820621210>3.0.CO;2-D 3056604

[24] Meyers MO Klauber-Demore N Ollila DW Amos KD Moore DT Drobish AA. Impact of breast cancer molecular subtypes on locoregional recurrence in patients treated with neoadjuvant chemotherapy for locally advanced breast cancer. Ann Surg Oncol (2011) 18(10):2851–7. doi: 10.1245/s10434-011-1665-8 21442348

[25] Cortazar P Zhang L Untch M Mehta K Costantino JP Wolmark N. Pathological complete response and long-term clinical benefit in breast cancer: the CTNeoBC pooled analysis. Lancet (2014) 384(9938):164–72. doi: 10.1016/S0140-6736(13)62422-8 24529560

